# Early phase of pupil dilation is mediated by the peripheral parasympathetic pathway

**DOI:** 10.1152/jn.00401.2021

**Published:** 2021-12-01

**Authors:** Chinatsu Marumo, Tamami Nakano

**Affiliations:** ^1^Faculty of Medicine, Osaka University, Osaka, Japan; ^2^Graduate School of Frontiers Bioscience, Osaka University, Osaka, Japan

**Keywords:** autonomic system, iris dilator muscle, iris sphincter muscle, pupil dilation, pupillometry

## Abstract

Pupil diameter fluctuates in association with changes in brain states induced by the neuromodulator systems. However, it remains unclear how the neuromodulator systems control the activity of the iris sphincter (constrictor) and dilator muscles to change the pupil size. The present study compared temporal patterns of pupil dilation during movement when each muscle was pharmacologically manipulated in the human eye. When the iris sphincter muscle was blocked with tropicamide, the latency of pupil dilation was delayed and the magnitude of pupil dilation was reduced during movement. In contrast, when the iris dilator muscle was continuously stimulated with phenylephrine, the latency and magnitude of rapid pupil dilation did not differ from the untreated control eye, but sustained pupil dilation was reduced until the end of movement. These results suggest that the iris sphincter muscle, which is under the control of the parasympathetic pathway, is quickly modulated by the neuromodulator system and plays a major role in rapid pupil dilation. However, the iris dilator muscle receives signals from the neuromodulator system with a slow latency and is involved in maintaining sustained pupil dilation.

**NEW & NOTEWORTHY** By pharmacologically manipulating the pupil dilator and constrictor muscles of human eye separately, we found that the pupil constrictor muscle is a primary controller of rapid pupil dilation upon brain arousal. However, the pupil dilator muscle, which is innervated by the sympathetic nervous system and is generally considered as a major regulator of pupil dilation, is not involved in rapid pupil dilation, but was involved in long-lasting pupil dilation.

## Graphical abstract

**Figure:**
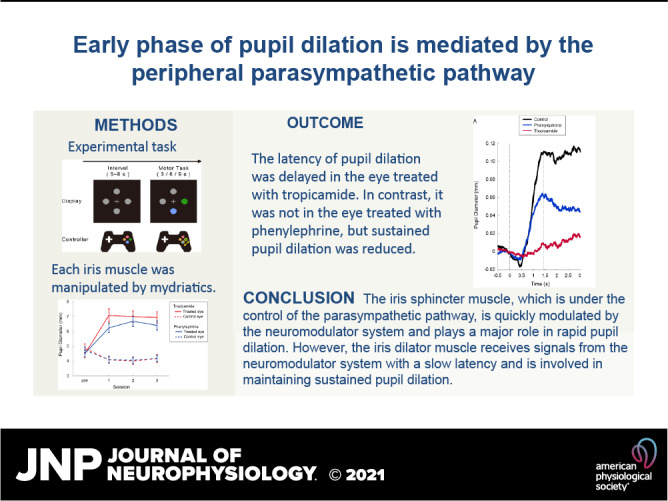

## INTRODUCTION

The pupil diameter of our eyes continuously fluctuates with changes in not only luminance and accommodation but also with various brain states related to arousal, locomotion, emotion, attention, and cognitive loads (1, 2). Although the precise neural pathways by which these changes in brain state are coupled to pupil size remain unclear, it has been suggested that these changes are modulated by the activities of neuromodulatory systems (3). In particular, locus coeruleus (LC) noradrenaline neurons are proposed to be the core region that induces pupil dilation (2). The monkey electrophysiological study reported that neural activation in the LC precedes pupil dilation, and the electronical stimulation in this site induces pupil dilation (4). The activities of noradrenergic projections to the cortex also increase in association with pupil dilation in mice (5). Consistently, the brain imaging study on humans reported that neural activity in the LC is positively correlated with pupil diameter (6). In addition to the LC-noradrenergic neuromodulatory system, several studies on mice have reported that activity in the cholinergic neuromodulatory system is also correlated with pupil dilation (5, 7). Based on these findings, pupil dilation has been widely used as a reliable indicator of cortical arousal modulated by the release of noradrenaline and acetylcholine.

The pupil size is controlled by the iris sphincter muscle and the iris dilator muscle, which constricts and dilates the pupil, respectively (8). The iris sphincter muscle is controlled by the Edinger–Westphal nucleus (EWN) and is under the control of the parasympathetic nervous system, whereas the iris dilator muscle is controlled by the superior cervical sympathetic ganglion and is under the control of the sympathetic nervous system. Therefore, it is widely accepted that pupil dilation accompanied by cortical arousal is mediated by the pupil dilator muscle. However, several studies reported that inhibition of the iris sphincter muscle activity by the eye drops of the cholinergic antagonist on human eyes altered pupil dilation due to mental effort, whereas inhibition of the pupil dilator muscle activity did not (9, 10). This suggests that the inhibitory input from the LC to the EWN or superior colliculus (SC) might induce pupil dilation via relaxation of the iris sphincter muscle. This raises the question of whether the sympathetically innervated dilator muscle is not involved in pupil dilation associated with cortical arousal, contrary to the commonly accepted theory.

To address this question, we focused on the previous finding that noradrenaline and acetylcholine activities in the cortex correlate with changes in pupil fluctuations at different time courses in mice (5). Rapid pupil dilation is highly associated with phasic activity in noradrenergic projections, whereas long-lasting pupil dilation during locomotion is accompanied by tonic activation in cholinergic projections. As various neuromodulator systems are involved in pupil dilation in different temporal patterns, we speculate that the iris sphincter and dilator muscles may be involved in pupil dilation at different time courses. To test this hypothesis, the present study examined how pupil dilation changes during movement of various durations when the autonomic inputs to the iris sphincter and dilator muscles were pharmacologically manipulated in humans. Since the previous study on mice (5) examined pupil dilation associated with locomotion, this study used the motor task for pupil dilation in humans in line with that study.

## METHODS

### Participants

Sixteen healthy volunteers participated in the experiments. All participants had normal vision—either uncorrected or corrected by contact lenses. The chosen sample sizes were similar to those in previous publications related to pupil response (9, 10). Two participants were excluded from the analysis because of the poor effect of mydriatic drops (*n* = 1) and serious measurement noise in the data (*n* = 1). The final sample of the pharmacological experiment consisted of 14 participants (mean age: 21.4 yr, range: 19–24 yr; 6 women). None of the participants had ocular or central nervous system pathologies, diabetes, or cardiovascular problems. In addition, an ophthalmologist confirmed that the anterior chamber angles were not narrow before the experiment. Before participation, we tested which eye, left or right, was the nondominant eye for each participant. The participants pointed out a visual marker with their index finger, and examined whether the position of the finger was shifted from the visual marker when they alternately covered either eye. When the finger was misaligned, the eye on the uncovered side was determined to be the nondominant eye. The review board of Osaka University approved the experimental protocol (FBS2019-1.48), and our procedures followed the guidelines outlined in the Declaration of Helsinki. All participants provided written informed consent before participating in the experiment.

### Pharmacological Treatment

In the pharmacological treatment, two kinds of mydriatic drops [two drops of phenylephrine 5% (Neosynesin) or one drop of tropicamide 0.4% (Mydrin-M)] were administered to the participants’ eyes on different days. Tropicamide, a cholinergic antagonist, was used to block signal transmission at the neuromuscular junctions of the iris sphincter muscle. Phenylephrine, an adrenergic α-1 receptor agonist, was used to contract the pupil dilator muscles. Since the adrenergic receptors at the neuromuscular junction are already activated by the adrenergic agonist, any changes in the release level of adrenaline are not reflected in the pupil diameter. We did not use an adrenergic antagonist because adrenergic antagonists for human eye drops are currently unavailable. Both phenylephrine and tropicamide are commonly used to widen the pupil during ophthalmic examinations. These mydriatic drops were administered at the same dosage as that used for regular ophthalmic examinations. Until mydriasis was recovered, the participant was advised to refrain from performing dangerous mechanical operations, such as driving a car, and looking directly at the sun or strong light. Pharmaceutical companies reported no side effects or after effects associated with the administration of these mydriatic drops; thus, participants had no particular disadvantage except the time they spent participating.

Before the instillation of the eye drop, the participants performed a short version of the experimental session in which they participated after instilling (presession). Subsequently, the mydriatic drop was instilled into the nondominant eye, and the participants were asked to close their eyes for 1 min. It usually takes 40 min for the phenylephrine to be fully effective, and 20 min for the tropicamide to be fully effective. To saturate the effect of the drugs fully, all experiments were performed after waiting for 40 min after instillation. The participants then performed three experimental sessions with a 5-min break between them. The order of instillation of the two drugs was counterbalanced across the participants. The washout period was at least 2 days. The drugs were instilled in the nondominant eye, and the dominant eye was used as the control eye. Ten out of 14 participants had their vision corrected by wearing contact lenses; the contact lens on the nondominant eye was removed throughout the experiment.

### Stimuli and Procedures

Participants sat in a chair in front of a 24-in. liquid crystal display (1,920 × 1,080 pixels, refresh rate 60 Hz, FlexScan, Eizo, Japan) and stabilized their head position using a chin rest and a forehead rest placed at a distance of 56 cm from the screen. They were asked to fixate on a white cross at the center of the screen (the size of cross 1°) throughout the experiment. The participants held a gamepad (Microsoft Sidewinder) and were requested to hit buttons with their right hand as much as possible while the visual cue was presented.

**Figure 1.**
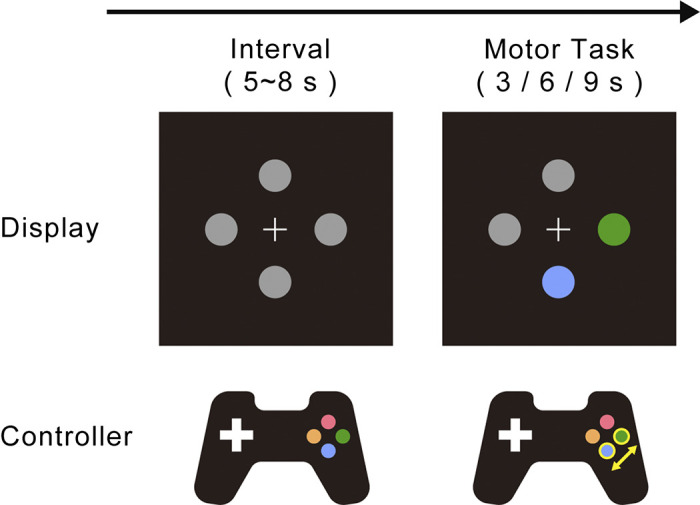
Experimental procedures. Four gray disks surrounding the fixation cross appeared in a display monitor. While two of the disks changed color for 3, 6, or 9 s, participants alternately keep hitting the buttons of the controller corresponding to them.

Four gray disks around a central white fixation cross were presented on the display monitor throughout the experiment (disk diameter: 1.2°; luminance: 14.5 cd/m^2^; fixation width: 1.0°, 1.48 cd/m^2^; background color: dark gray, 0.2 cd/m^2^; distance between the fixation point and the disks: 4°) (Fig. 1). Each trial started when two of the four disks, selected randomly, changed color (onset of visual cue). The color of each disk on the display corresponded to the color of the four buttons of the gamepad (red disk: 10.2 cd/m^2^, *x* 0.65, *y* 0.33; blue disk: 2.3 cd/m^2^, *x* 0.15, *y* 0.04; green disk: 20.2 cd/m^2^, *x* 0.30, *y* 0.61; yellow disk: 15.8 cd/m^2^, *x* 0.43, *y* 0.51). The participants were instructed to continue hitting the colored buttons alternately until the colors of the two disks turned gray. Each session consisted of 10 trials of 3, 6, or 9 s each (a total of 30 trials). The intertrial interval was set to a random duration, between 5 and 8 s.

Participants first performed a short version of the experimental session (pre-session, 6 trials for each duration, a total of 18 trials). After pharmacological instillation, they performed three sessions with a 5-min break between them. During the experiment, the pupil areas of both eyes were monitored using a near-infrared eye tracker (Eyelink II, SR Research) at a sampling frequency of 250 Hz.

### Data Analysis

All data analyses were conducted using MATLAB (2020b, MathWorks, Inc.). To directly compare the increase in pupil size between the treated (dilated) and untreated (undilated) eyes, the pupil area (mm^2^) was converted to the pupil diameter (mm) by taking the square root of the pupil area multiplied by 4/π. We also smoothed data by simply moving the average over a sliding window of 20 ms. Next, to exclude an artifact due to eye blinks on the pupil size, we first detected the blink onset and offset time by finding a pair of rapid decreases and increases within 500 ms in the pupil diameter (11). Because the artifact caused by blinking appeared before and after the detected time, the data from 40 ms before blink onset time to 80 ms after blink offset time was set as a missing value. The pupil response was extracted from 1 s before the onset of the visual cue to 1 s after the offset of the visual cue for each trial, and was adjusted using the average pupil response 0.1 s before onset of the visual cue. The time series of mean pupil size across each condition was calculated for each participant and used in the subsequent statistical analysis. We conducted a statistical test on these values using a three-way analysis of variance (ANOVA) with factors of time phase (early/late), ocular instillation (treated/untreated eyes), and duration (3/6/9 s) for each eye drop (tropicamide/phenylephrine).

The latency of pupil dilation was measured for each eye in each participant using the extrapolation method (12), in which a line through the points at 25% and 75% of the peak value was extrapolated to intersect the line representing the value of 0. The time at the point of intersection was considered to be the latency of pupil dilation. The distribution of the latency was statistically compared between the eye types using one-way ANOVA.

## RESULTS

**Figure 2.**
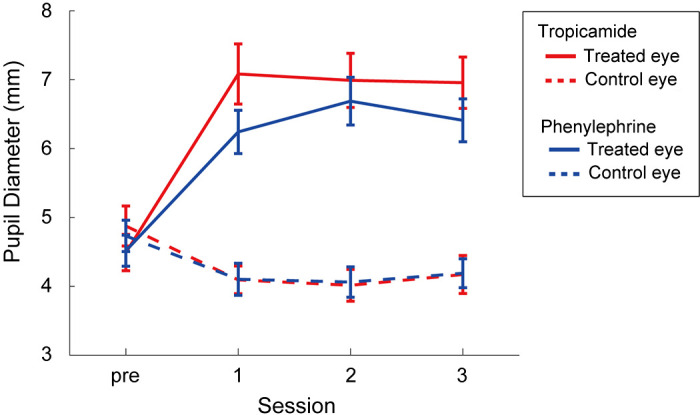
Pupil diameter changes in treated and control eyes after the pharmacological treatment. The first session was conducted 40 min after the instillation. Subsequently, the second and third sessions were conducted with 5-min break. The red and blue lines represent the eye instilled by tropicamide and phenylephrine, respectively. The error bars represent standard errors.

In this experiment, we compared pupil dilation during movement between drug conditions (tropicamide/phenylephrine). We first analyzed the absolute values of the pupil diameter before and after instillation to confirm the effects of the mydriatic drugs (Fig. 2). Before instillation, the average pupil diameter of the treated and control eyes was almost the same in both drugs (tropicamide-treated eye: 4.5 ± 0.9 mm, control eye, 4.9 ± 1.1 mm; phenylephrine-treated eye: 4.5 ± 0.8 mm, control eye, 4.7 ± 0.8 mm). However, 40 min after instillation, the pupil diameter of the eyes that received mydriatic drugs expanded to more than 1.5 times as much as before instillation, whereas the pupil diameters of the untreated eyes tended to shrink slightly. This tendency continued across three experimental sessions (the last session was completed within 80 min after instillation). The average pupil diameter of the eye treated with tropicamide for each session was 7.1 ± 1.6 mm, 7.0 ± 1.4 mm, and 7.0 ± 0.13 mm, respectively, and that of the eye treated with phenylephrine was 6.2 ± 1.1 mm, 6.7 ± 1.2 mm, and 6.4 ± 1.1 mm, respectively. The three-way ANOVA with factors of drug type (tropicamide and phenylephrine), instillation (treated and untreated eyes), and session (pre, 1st, 2nd, and 3rd) revealed a significant interaction of three factors (*F*_3,39 _= 43.31, *P* = 0.03). The mean pupil size of the treated eye was significantly larger than that of the untreated eye at 1st, 2nd, and 3rd sessions by the instillation of both tropicamide (*F*_1,104_ = 76.05, 75.32, and 65.99, respectively, *P* < 0.001) and phenylephrine (*F*_1,104 _= 38.93, 58.68, and 41.92, respectively, *P* < 0.001). On the contrary, at presession (before instillation of drugs), mean pupil size did not differ between the treated and untreated eyes after the instillation of tropicamide (*F*_1,104 _= 1.27, *P* = 0.26) and phenylephrine (*F*_1,104 _= 0.39, *P* = 0.53). In addition, the mean pupil size of the tropicamide-treated eye was significantly larger than that of the phenylephrine-treated eye at 1st session (*F*_1,104_ = 7.02, *P* = 0.009). These results clearly demonstrate the effect of mydriatic drugs on treated eyes.

**Figure 3.**
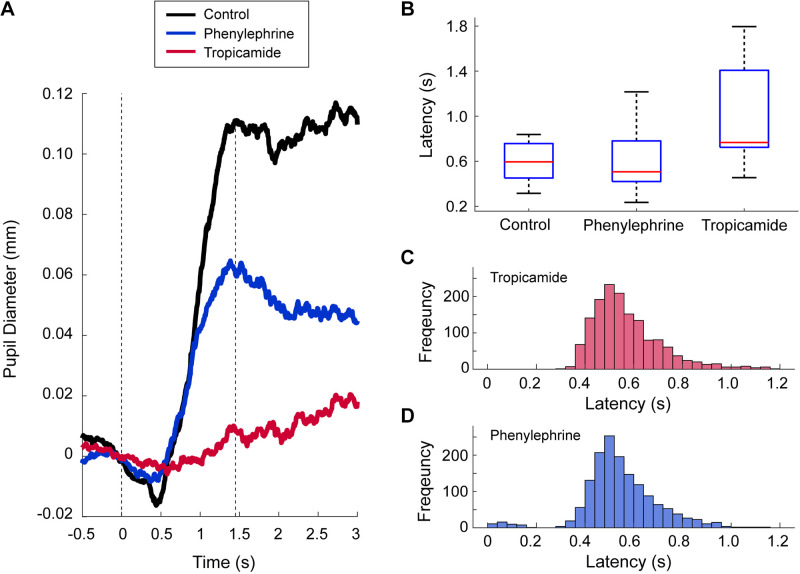
Latency of pupil dilation and movement. *A*: the averaged time courses of pupil size across all trials at around the time of onset of the visual cue. The black line represents the pupil size of the intact control eye. The blue and red lines represent the pupil size of the eye instilled by phenylephrine and tropicamide, respectively. The *left* dotted line represents the onset of the visual cue. The *right* dotted line represents the initial time of peak pupil dilation in the control eye. *B*: comparison of the latency of pupil dilation between treated and untreated eyes. In each rectangle, the central mark is the median, the edges of the box are the 25th and 75th percentiles, and the whiskers extend to the minimum and maximum data points. *C*: histogram of the latency of hand movement in the experiments with tropicamide treatment. *D*: histogram of the latency of hand movement in the experiments with phenylephrine treatment.

Next, we compared the time course of relative pupil size at the initial phase in the trials (first 3 s after the onset of the trial) between the treated and untreated eyes for each drug across all trials (Fig. 3*A*). The pupil size of the control intact eyes increased rapidly after the onset of the visual cue, with a latency of 0.62 s, and reached an initial peak at 1.48 s after the onset of the trial. The pupil size of the eye treated with phenylephrine increased with a latency of 0.54 s, similar to that of the control eyes (blue line in Fig. 3*A*). In contrast, the eye treated with tropicamide showed an increase in pupil size with a latency of 0.85 s (red line in Fig. 3*A*). We further measured the latency of pupil dilation in each participant and compared its distribution among the three different treatments (Fig. 3*B*). Because some participants showed no apparent increase during the initial phase, the number of latencies successfully detected for control, phenylephrine, and tropicamide were 12, 11, and 10, respectively. As shown in Fig. 3*B*, the mean latency of pupil dilation in the eye treated with tropicamide was much longer than that in the eye treated with phenylephrine or the intact control eye (tropicamide: 0.96 ± 0.45 s; phenylephrine: 0.59 ± 0.28 s; control: 0.60 ± 0.18 s). One-way ANOVA revealed a significant main effect of the drug condition (*F*_2,30 _= 4.58, *P* = 0.018), and post hoc Ryan test confirmed a significant difference between tropicamide and phenylephrine (*t*_30_ = 2.64, *P* = 0.013) and between tropicamide and control (*t*_30_ = 2.66, *P* = 0.012), whereas there was no difference between phenylephrine and control (*t*_30_ = 0.026, *P* = 0.98).

We also analyzed the latency of movement in response to the visual cue. In response to the color change of the target disks (the onset of trial), the participants started hitting the button with a latency of ∼0.56 s (tropicamide: 0.57 ± 0.09 s, Fig. 3*C*; phenylephrine: 0.55 ± 0.08 s, Fig. 3*D*). Therefore, the latency of pupil dilation in the control eye was almost the same as the latency of movement.

**Figure 4.**
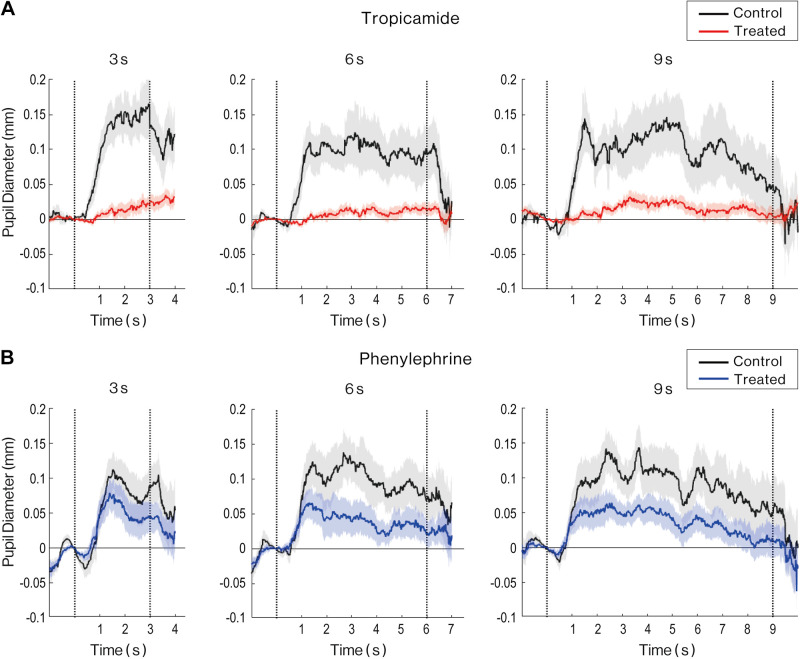
Comparison of the effect of pharmacological treatment on the time course of pupil size during motor response. *A*: the time course of pupil size in the eye treated with tropicamide (red line) and the intact control eye (black) for each duration. *B*: the time course of pupil size in the eye treated with phenylephrine (blue line) and the intact control eye (black) for each duration. The shaded areas represent standard errors across participants.

We then compared the temporal changes in pupil size between treated and untreated eyes for each trial length and for each drug (Fig. 4). For both drugs, the pupil size in the control intact eyes increased rapidly after the onset of the trial and remained large until the end of the trial, regardless of the length of the trial (black lines in Fig. 4, *A* and *B*). However, the contralateral eye that received the tropicamide showed a delayed increase in pupil size, and the change in pupil size remained much smaller than in the control eye until the end of the trial (red lines in Fig. 4*A*). The eyes treated with phenylephrine showed different patterns of pupil change. After the onset of trial, the pupil size of the treated eyes showed an increase similar to that of the control eyes. After more than 1 s, it decreased to about half that of the control eye, which continued until the end of the trial (blue lines in Fig. 4*B*). The same tendency was consistently observed among the participants. The typical response of one participant is shown in Supplemental Fig. S1 (all Supplemental Material is available at https://doi.org/10.6084/m9.figshare.16922458).

**Figure 5.**
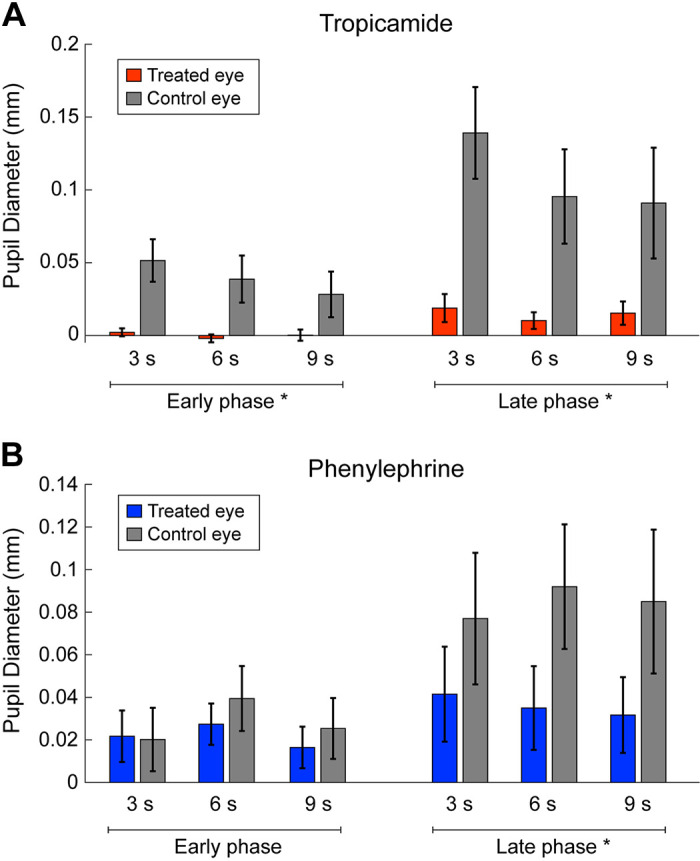
Comparison of pupil size between early and late phases. The red and blue bars represent the mean pupil sizes instilled by tropicamide (*A*) and phenylephrine (*B*), respectively. The gray bars represent those of the untreated eye. The error bars represent standard error among participants; *significant difference between treated and control eyes.

Therefore, we compared the average change in pupil size between treated and control eyes for each drug in the early phase (0.5–1.48 s after the onset of trial) and the late phase (from 1.48 s after the onset of the trial until its offset). The time window of the early phase was determined based on the latency and the initial time of peak pupil dilation in the control eye (Fig. 3*A*). Tropicamide suppressed pupil dilation in both phases, but especially in the early phase (Fig. 5*A*). The three-way ANOVAs with factors of phase (early/late), task duration (3/6/9 s), and instillation (treated/control eyes) detected a significant main effect of instillation (*F*_1_,_13 _= 13.02, *P* = 0.003) and a significant interaction of phase and duration (*F*_2_,_26_ = 6.05, *P* = 0.007). The post hoc Ryan test confirmed that the change in pupil size for 3 s was significantly greater than that for 6 s and 9 s in the late phase (*t*_52_ = 3.75, *P* = 0.0005, *t*_52_ = 3.85, *P* = 0.0005). Phenylephrine, however, suppressed pupil dilation only in the late phase (Fig. 5*B*). The three-way ANOVA detected a significant interaction between phase and instillation (*F*_1_,_13 _= 11.8, *P* = 0.004). The post hoc test reveals that instillation of phenylephrine significantly affects the pupil dilation in the late phase (*F*_1_,_26 _= 25.26, *P* < 0.001).

## DISCUSSION

The present study confirmed that the arousal of pupil response to the movement task consisted of two phases. In the early phase, the pupil size rapidly increased accompanied with the initiation of movement, and in the late phase, the pupil dilations were maintained until the end of the movement. This rapid pupil dilation in the early phase was delayed and reduced in the eye instilled with tropicamide, whereas it did not change in the eye instilled with phenylephrine. In contrast, long-lasting pupil dilation during movement was suppressed in eyes instilled with tropicamide or phenylephrine. These results suggest that the iris sphincter muscle plays a primary role in controlling rapid pupil dilation with the onset of movement, whereas the iris dilator and iris sphincter muscles are involved in sustained pupil dilation during movement.

Previous studies using the pharmacological blocking method in human eyes reported that pupil dilation caused by cognitive effort is altered only when the iris sphincter muscle is blocked (9, 10). In contrast, the present study demonstrated that the iris dilator muscle also contributes to sustained pupil dilation during movement, but the initial phase of pupil dilation due to changes in brain state is mainly induced by relaxation of the iris sphincter muscle. Pupil dilation is widely accepted to be caused by the activation of noradrenergic and cholinergic neuromodulatory systems in the central nervous system (1, 2). If so, how do they quickly influence the activity of the iris sphincter muscle, which is controlled by the peripheral parasympathetic pathway? One response could be the inhibitory input via noradrenaline α 2 receptors from the LC noradrenaline neurons to the EWN. Considering that the EWN is the center of the pupillary light reflex and the quick control of the activity of the iris sphincter muscle, it is plausible that pupil dilation occurs at short latencies due to the direct action of the neuromodulator system on the EWN. Another response could be the input from the SC to the EWN (13). The SC receives input from the LC neurons, and neural activity in the SC also precedes pupil dilation (4). Considering that the SC is deeply involved in orienting and eye movement, this pathway can also induce rapid pupil dilation. Further investigation measuring neural activity on the EWN and SC is necessary to identify which neural pathway regulates rapid pupil dilation due to changes in brain state.

Another important finding in the present study is that the iris dilator muscle has a longer latency than the iris sphincter muscle as a contributor to the observed pupil dilation. This suggests that signal transmission from the neuromodulatory systems to the dilator muscle is more indirect than that from the iris sphincter muscle. The iris dilator muscle is under the control of the sympathetic nervous system and is controlled by the hypothalamus via the superior cervical sympathetic ganglion. The hypothalamus receives input from various neuromodulator systems, including the LC noradrenaline neurons and basal forebrain cholinergic neurons (14, 15). We assume that this hypothalamus-mediated control of pupil diameter takes longer to act than the EWN-mediated control of pupil diameter because multiple neural sites, including the spinal cord, are involved in signal transmission. Interestingly, a previous study using mice reported that the activity of noradrenaline projections in the cortex tracks the initial changes in pupil dilation after the onset of locomotion, whereas cholinergic projections remain active throughout pupil dilation during locomotion (5). Given that the iris dilator muscle is involved in sustained pupil enlargement until the end of the movement, it is possible that the cholinergic neuromodulator system is mainly involved in controlling the iris dilator muscle via the hypothalamus. Further studies are needed to clarify the neural pathways between the iris dilator muscle and neuromodulator systems.

One might argue that the reduced change in pupil size in the eye treated with tropicamide might derive from the ceiling effect on pupil size. Because we did not measure maximum pupil size, we do not know whether the pupil could have dilated further. However, numerous studies have reported that the combination of phenylephrine and tropicamide is much more effective on mydriasis than either drug alone (16, 17), and that the iris sphincter and dilator muscles are independently involved in mydriasis and pupil dilation does not reach a maximum unless both muscles are pharmacologically manipulated at the same time. We, therefore, assume that the decreased pupil response of the eye treated with tropicamide does not derive from a ceiling effect. Further studies are expected to examine the capability of iris dilation by administering both drugs simultaneously.

There are several limitations in the present study. First, the brightness of the target color might have affected pupil size because a change in luminance can potentially induce a pupillary light reflex. However, the latency of pupil dilation in the current study (0.55 s) was much longer than the latency of the pupillary light reflex (0.22 s) (18). In addition, the direction of change in brightness was not biased toward inducing pupil dilation. We, therefore, assume that the brightness change is unlikely to have substantially affected the pupil response. Nonetheless, it is important to verify the present result using a stimulus that does not cause a change in luminance.

Second, previous studies have reported that pupil size displays a slow oscillatory fluctuation, known as a hippus, which diminishes when the iris sphincter muscle is pharmacologically blocked (19, 20). In the present study, we focused on the latency and magnitude of change in pupil size, and we did not analyze oscillatory activity because artifacts caused by frequent blinking and measurement noises can affect frequency information. Further studies are needed to examine oscillatory fluctuations of pupil size in association with the arousal response when the autonomic pathway is pharmacologically manipulated.

Pupillometry is widely used as a measure of brain state, which is altered by the activity of noradrenergic and cholinergic neuromodulator systems. However, the neural pathways by which these neuromodulator systems induce changes in pupil size remain unclear. The present study revealed that the iris sphincter and dilator muscles are involved in pupil dilation with different temporal patterns. This can be a clue to identify the neural pathways linking neuromodulator systems and pupil dilation.

## SUPPLEMENTAL DATA

Supplemental Fig. S1: https://doi.org/10.6084/m9.figshare.16922458.

## GRANTS

This work was supported by a Grant-in-Aid 18H04084, 18H05522 awarded to T.N. from the Ministry of Education, Culture, Sports, Science and Technology, Japan, as well as a PRESTO Grant, “The Future of Humans and Interactions” No. 30227, awarded to T.N. from the Japan Science and Technology Agency, Japan.

## DISCLOSURES

No conflicts of interest, financial or otherwise, are declared by the authors.

## AUTHOR CONTRIBUTIONS

T.N. conceived and designed research; C.M. performed experiments; C.M. and T.N. analyzed data; C.M. prepared figures; C.M. and T.N. drafted manuscript; C.M. and T.N. approved final version of manuscript.
